# Immune imbalance in Lupus Nephritis: The intersection of T-Cell and ferroptosis

**DOI:** 10.3389/fimmu.2024.1520570

**Published:** 2024-12-12

**Authors:** Yunhe Fan, Kuai Ma, Yumeng Lin, Junyi Ren, Haoyu Peng, Lan Yuan, Moussa Ide Nasser, Xuan Jiang, Ke Wang

**Affiliations:** ^1^ School of Medical and Life Sciences, Chengdu University of Traditional Chinese Medicine, Deyang Hospital Affiliated Hospital of Chengdu University of Traditional Chinese Medicine, Deyang, China; ^2^ Department of Nephrology, Osaka University Graduate School of Medicine, Osaka, Japan; ^3^ Health Management Center, Nanjing Tongren Hospital, School of Medicine, Southeast University, Nanjing, China; ^4^ University of Electronic Science and Technology of China, School of Medicine, Chengdu, China

**Keywords:** ferroptosis, T cells, Lupus Nephritis, immune imbalance, cellular metabolism

## Abstract

Ferroptosis is a novel form of cell death characterized by unlimited accumulation of iron-dependent lipid peroxides. It is often accompanied by disease, and the relationship between ferroptosis of immune cells and immune regulation has been attracting increasing attention. Initially, it was found in cancer research that the inhibition of regulatory T cell (Treg) ferroptosis and the promotion of CD8+ T cell ferroptosis jointly promoted the formation of an immune-tolerant environment in tumors. T-cell ferroptosis has subsequently been found to have immunoregulatory effects in other diseases. As an autoimmune disease characterized by immune imbalance, T-cell ferroptosis has attracted attention for its potential in regulating immune balance in lupus nephritis. This article reviews the metabolic processes within different T-cell subsets in lupus nephritis (LN), including T follicular helper (TFH) cells, T helper (Th)17 cells, Th1 cells, Th2 cells, and Treg cells, and reveals that these cellular metabolisms not only facilitate the formation of a T-cell immune imbalance but are also closely associated with the occurrence of ferroptosis. Consequently, we hypothesize that targeting the metabolic pathways of ferroptosis could become a novel research direction for effectively treating the immune imbalance in lupus nephritis by altering T-cell differentiation and the incidence of ferroptosis.

## 1 Introduction

The key mechanism of ferroptosis involves the upregulation of lipid peroxidation pathways driven by iron, reactive oxygen species (ROS), and polyunsaturated fatty acids (PUFAs), coupled with the downregulation of antioxidant mechanisms, primarily selenium-dependent glutathione peroxidase 4 (GPX4) (1). Increasing evidence suggests that modulating metabolic targets involved in T-cell ferroptosis can alter immune imbalances. Initially, this regulatory mechanism attracted attention in the context of tumor immunomodulation. Ferroptosis in Treg cells is often suppressed by upregulated antioxidant mechanisms, which contribute to tumor immune evasion (2). However, ferroptosis in CD8+ T cells is frequently promoted by increased lipid synthesis, thereby reducing their capacity for immune recognition and killing of tumor cells (3). Notably, altering T-cell ferroptosis-related metabolic targets, such as promoting ferroptosis in Treg cells or inhibiting ferroptosis in CD8+ T cells, has been shown to improve cancer prognosis. Therefore, given that T-cell ferroptosis is a promising disease immunoregulatory mechanism, its potential therapeutic role in other diseases has also garnered significant interest (4).

Systemic lupus erythematosus (SLE) is characterized by immune tolerance disorders and hyperactivity of immunological reactivity, leading to immune imbalance (5). LN is a common complication and cause of death in SLE patients (6). Recent studies have shown that T-cell immune imbalance is the key pathogenesis of LN (7, 8). Specifically, upregulated effector T (Teff) cells contribute to kidney injury through the formation of a proinflammatory environment and the promotion of fibrosis (9). By assisting in humoral immunity, TFH cells produce more pathogenic antibodies, exacerbating autoimmune inflammation (10). Additionally, the downregulation of Treg cells in LN fails to maintain immune tolerance, allowing the adaptive immune system to no longer protect self-antigens while recognizing and eliminating pathogens and accelerating kidney injury (11). Although T-cell immune imbalance is crucial in LN, little is known regarding its regulation. Therefore, research on the mechanisms by which T-cell ferroptosis regulates T-cell immune imbalance in the LN has attracted significant attention.

In this review, we first described the unique manifestations of immune imbalances among various T-cell subsets in LNs. We highlighted that metabolic alterations in T cells within the LN not only promote the aberrant differentiation of T cells, leading to immune dysregulation but also variably augment the occurrence of ferroptosis across different T-cell subsets. Similarly, we revisited the current understanding of how the regulation of metabolic targets related to ferroptosis in these T-cell subsets could modulate immune imbalances in LN. We emphasized the potential of metabolic enzymes and molecules associated with ferroptosis as future therapeutic targets for treating immune imbalances in LN.

## 2 The mechanism of Ferroptosis

**Figure 1:**
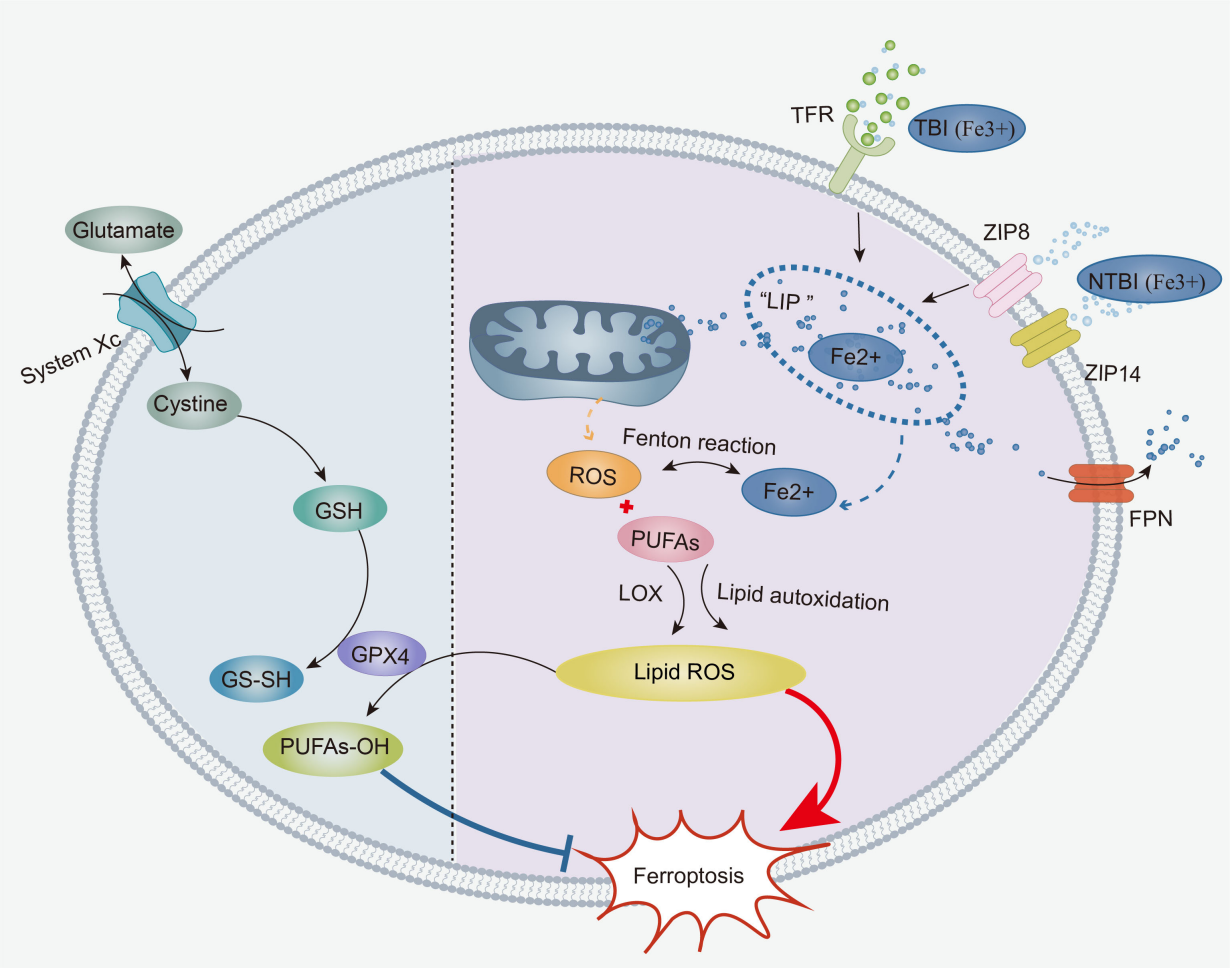
The core mechanisms of ferroptosis: The accumulation of labile iron, ROS, and PUFAs in cells contributes to the onset of ferroptosis. TBI and NTBI enter the cell and are temporarily stored in an “iron pool”. Subsequently, labile iron from the “iron pool” initiates a Fenton reaction that produces an abundance of ROS, leading to an increase in intracellular ROS. Under the effect of LOXs, intracellular ROS first undergoes enzymatic lipid peroxidation with PUFAs, generating lipid ROS. Once a critical threshold is reached, intracellular ROS directly triggers non-enzymatic lipid autoxidation with membrane-bound PUFAs, resulting in a large amount of lipid ROS. Furthermore, the weakening of the xCT-GPX4 antioxidant system promotes ferroptosis. Cystine is transported into the cell via System xCT, participating in the synthesis of reduced GSH. Then, reduced GSH acts on GPX4 to reduce lipid ROS to lipid alcohols, thereby inhibiting the production of lipid ROS. ROS, Reactive Oxygen Species; PUFAs, Polyunsaturated Fatty Acids; TBI, Transferrin-bound Iron; NTBI, Non-Transferrin-Bound Iron; TFR, Transferrin Receptors; Non-Transferrin Receptors, ZIP8, ZIP14; LOXs, Lipoxygenases; GSH, glutathione.

The continuous accumulation of unstable iron, increase of ROS, and supply of PUFAs in cells, coupled with weakened antioxidant mechanisms, lead to an unlimited increase in lipid ROS in cells, which is the key factor that induces ferroptosis (1). Among them, weakened antioxidant mechanisms are closely related to glutathione metabolism and the GPX4 enzyme. Therefore, abnormal metabolism in cells is often the key to identifying ferroptosis. In addition, many studies have shown that mitochondria usually exhibit atrophic and dense morphology during ferroptosis, which is also key evidence for verifying the occurrence of ferroptosis (12). A deeper understanding of metabolic changes in ferroptosis is highly important for identifying the occurrence of T-cell ferroptosis in diseases (**Figure 1**).

### 2.1 Accumulation of cellular iron

Once iron enters the bloodstream from different sources in the human body, it must undergo absorption, use, storage, and excretion by cells. Cellular iron absorption involves the uptake of transferrin-bound iron (TBI) into the cell via traditional transferrin receptor 1 (TfR-1, also known as CD71) (13), as well as the uptake of nontransferrin-bound free iron (NTBI), mostly by liver cells (14). Upon entering the cytoplasm, TBI and NTBI are transiently held in the cytoplasm’s “labile iron pool” (LIP) in an unstable ferrous state. Iron in the LIP is then weakly attached and carried to the mitochondria for use. Iron inside mitochondria is a crucial component for the production of heme and Fe-S clusters, which are involved in a variety of cellular metabolic processes (15, 16). The majority of unutilized iron is stored inside the cell as ferritin (17). Excess iron is specifically linked to ferritin by the iron chaperones poly r(C)-binding protein (PCBP) 1 and PCBP 2 (18, 19) and then transported out of the cell exclusively by ferroportin (FPN)-1 (20). Furthermore, the iron content in the unstable iron pool inside the cell is carefully regulated by iron regulators and iron regulatory proteins. These proteins control the release and storage of iron in the cell to prevent it from exceeding 5% of the total iron content and maintain a balanced iron level within the cell (17, 21, 22). Disruption of iron metabolism may result in the aberrant buildup of iron inside the cell, which can lead to ferroptosis. Studies have shown that the process of autophagy, which involves the breakdown of ferritin (a protein that stores iron), may lead to the build-up of unstable iron in cells and facilitate ferroptosis (23–25). Disruption of the main receptor TfR1, which is responsible for cellular iron absorption, has been shown to successfully prevent ferroptosis (26). Facilitating the elimination of iron from inside cells has been scientifically shown to successfully prevent iron-induced cell death (27, 28).

### 2.2 ROS accumulation

ROS represent a collective term for oxygen-containing radicals and peroxides associated with oxygen metabolism in living organisms. ROS are characterized by unpaired electrons, rendering them highly chemically reactive. When they encounter nonradical species abundantly present in the body, they initiate electron-snatching chain reactions (29).

The ROS generated by the Fenton reaction are considered the primary source of ROS in ferroptosis. In LIP, divalent iron reacts with cytoplasmic hydrogen peroxide (H2O2) via the Fenton reaction, producing trivalent iron, OH−, and highly reactive hydroxyl radicals (OH•) (30, 31). OH• subsequently engages in electron-snatching chain reactions with cellular lipids, leading to the generation of toxic lipid ROS [also called lipid peroxides, LPOs or phospholipid hydroperoxide (PLOOH)].

Furthermore, numerous studies have shown that mitochondrial ROS (mtROS) (32) and nicotinamide adenine dinucleotide (phosphate) (NAD(P)H) oxidase (NOX)-derived ROS are significant electron predators involved in the generation of lipid ROS (33–35). Therefore, in addition to intracellular iron accumulation, the accumulation of ROS from multiple sources within cells, which are critical triggers for lipid ROS production, represents the second major factor in ferroptosis.

### 2.3 The occurrence of lipid peroxidation

Many studies have shown that PUFAs in the phospholipids of endoplasmic reticulum (ER) membranes, primarily arachidonic acid (AA) and adrenic acid (AdA), are key lipid substrates for the occurrence of lipid peroxidation (36–39). Acyl-CoA synthetase long-chain family member 4 (ACSL4) promotes the synthesis of PUFAs (40), whereas lysophosphatidylcholine acyltransferase 3 (LPCAT3) facilitates the esterification of PUFAs and their integration into membrane phospholipids (41).

PUFAs and ROS undergo a process of lipid peroxidation that involves both enzymatic and nonenzymatic stages. The enzymatic stage refers to the process by which PUFAs, under the action of lipoxygenases (LOXs), react with ROS to form lipid ROS (36, 42). Arachidonate lipoxygenase 15 (ALOX15) has been identified as the key enzyme in the enzymatic peroxidation of PUFAs (42–44). In addition, arachidonate lipoxygenase 12 (ALOX12) (45) and cytochrome P450 enzymes (CYPs) (46) have also been found to induce lipid peroxidation. The nonenzymatic stage occurs when intracellular iron accumulates, leading to Fenton reactions between intracellular ROS and membrane-bound PUFAs, thereby initiating lipid autoxidation (31, 47). This nonenzymatic lipid peroxidation plays a dominant role in the accumulation of lipid ROS during ferroptosis (42). Current research still debates the sequence of the enzymatic and nonenzymatic stages, but many studies support that once the enzymatically produced lipid ROS reach a critical threshold, they can trigger nonenzymatic lipid peroxidation (31, 47). Ultimately, lipid ROS first accumulate in the ER membrane and then in the cell membrane (38). Ferroptosis, a form of regulated cell death, occurs when the cell’s capacity to repair membrane damage is overwhelmed, leading to cell demise (42).

### 2.4 Antioxidant mechanisms

The strengthening of iron-dependent lipid peroxidation and the weakening of the antioxidant system are the third significant triggers for ferroptosis. Recent studies in cancer cells have demonstrated that altering the intracellular redox state can induce ferroptosis (48). Therefore, when an oxidative‒reductive imbalance occurs within a cell, vigilant monitoring of the potential induction of ferroptosis is essential.

#### 2.4.1 The xCT- GPX4 antioxidant system

In general, the xCT-GPX4 system is considered the primary antioxidant mechanism against ferroptosis. Its key components include cystine/glutamate antiporter solute carrier family 7 member 11 (SLC7A11), which is also known as xCT, and GPX4. Most cells initially take up cysteine (Cys) via xCT. Subsequently, cysteine is converted to cystine through the action of the reducing agent glutathione (GSH) or thioredoxin reductase 1 (TXNRD1), and it participates in GSH synthesis (47, 49). In mammals, TXNRD1 belongs to the thioredoxin reductase (TrxR) family and is also a selenoprotein. The Trx reductive system to which TXNRD1 belongs, along with the GSH reduction system, collaboratively eliminates ROS *in vivo*, maintaining the cellular redox balance (50). Through the action of GPX4, synthesized GSH subsequently reduces lipid ROS to lipid alcohols, thereby inhibiting ferroptosis (51–53). Therefore, the xCT-GPX4 system is a necessary antioxidant mechanism that directly targets the inhibition of lipid ROS generation. Studies have shown that GPX4 knockout cells exhibit high accumulation of ROS and lipid peroxidation products, providing further evidence for this mechanism (54). Notably, Gpx4, as a selenoprotein, requires the presence of selenium for its antioxidative function (51, 55).

Conversely, inhibition of the xCT‒GPX4 system can induce ferroptosis. On the basis of this mechanism, various ferroptosis inducers (FINs), such as buthionine sulfoximine (BSO) and erastin, have been developed and have garnered widespread attention in cancer therapy research (56–58). Notably, recent studies have suggested that ras-selective lethal 3 (RSL3), as a type of FINs, may act as an inhibitor of TXNRD1, not only GPX4. First, RSL3 increases ROS levels by inhibiting the TrX system, promoting the generation of lipid ROS. Second, it enhances the accumulation of lipid ROS by inhibiting GPX4, thereby increasing susceptibility to ferroptosis through a dual mechanism (53, 59). Furthermore, recent research has revealed a significant association between the anticancer mechanism of the tumor suppressor gene P53 and the inhibition of xCT-GPX4-induced ferroptosis. In contrast to FINs, P53 achieves its anticancer effects through the dual regulation of peroxidation and weakening antiperoxidation mechanisms. P53 downregulates SLC7A11 expression, directly inhibiting the xCT-GPX4 antioxidant system. However, P53 downregulation of SLC7A11 leads to the release of ALOX12 bound to it, promoting membrane lipid peroxidation and accelerating ferroptosis (45, 60). In summary, weakening of the xCT-GPX4 system, a critical intracellular antioxidant mechanism, is essential for inducing ferroptosis, and this mechanism holds promise for targeting various pathogenic cells in the treatment of various diseases.

In LN, there is a significant increase in oxidative stress due to the presence of autoantibodies, immune complexes, and cytokines such as interferon-α (61, 62). This oxidative stress leads to the depletion of antioxidants, including GPX4, making kidney cells more vulnerable to damage and death (61, 63). Lipidomic analyses of kidneys confirm excessive lipid peroxidation consistent with ferroptosis in LN (64). Ferroptosis, driven by GPX4 inhibition or dysfunction, is increasingly recognized as a mechanism of kidney cell death in LN (65, 66). This process exacerbates inflammation and tissue injury in the renal microenvironment (65, 67). The low expression of GPX4 in these kidneys correlates with tubular damage, highlighting its protective role against ferroptosis-induced injury (68).

Beyond its role in ferroptosis, GPX4 also influences immune cell function, particularly in regulating B cells and neutrophil, which are central to LN pathogenesis. Research indicates that LN is associated with attenuated expression of SLC7A11, this disrupts the antioxidant system, further reducing GPX4 activity and significantly enhances ferroptosis in B cells and reduces their proliferation (69). Research has found that in LN, GPX4 mechanisms involve autoantibodies and interferon-α in serum, which promote neutrophil ferroptosis by enhancing cAMP response element modulator (CREM)α binding to the GPX4 promoter (70). This binding reduces GPX4 expression and leads to an increase in autoantigens produced by neutrophil ferroptosis (70). Given the critical role of GPX4 in ferroptosis, further investigation into GPX4-mediated T cell ferroptosis and its regulation would be of great significance for understanding and potentially treating LN.

#### 2.4.2 The non xCT- GPX4 antioxidant systems

In addition to GPX4, ferroptosis suppressor protein 1 (FSP1, also known as AIFM2) and coenzyme Q10 (CoQ10, also referred to as ubiquinone) constitute the second major antioxidant system in ferroptosis. Unlike the xCT-GPX4 system, which combats oxidation by reducing already generated lipid ROS, the FSP1-CoQ10 system functions as an antioxidant by reducing the generation of lipid ROS, as described in the following mechanism. FSP1 utilizes NAD(P)H to reduce CoQ10 to its reduced form, Coenzyme Q10H2 (CoQ10H2, also known as ubiquinol), thereby consuming ROS and reducing the generation of lipid ROS (71, 72).

Dihydroorotate dehydrogenase (DHODH) shares a similar function with FSP1, as it can also reduce ubiquinone to ubiquinol, forming an antiferroptotic mechanism similar to that of the FSP1-CoQ10-CoQ10H2 system, known as the DHODH-CoQ10H2 system, to inhibit ferroptosis (73). The distinction between these two systems lies in their subcellular localization; FSP1 is primarily distributed throughout the plasma membrane and other nonmitochondrial membranes, whereas DHODH is mainly localized within the mitochondria (73). However, both systems work in coordination with their respective xCT-GPX4 systems to counteract the occurrence of lipid peroxides (74).

In the case of the GTP cyclohydrolase-1 (GCH1) -tetrahydrobiopterin (BH4) pathway, GCH1 is a critical enzyme in BH4 biosynthesis, and both play essential roles in ferroptotic antioxidant mechanisms. BH4 can promote the synthesis of CoQ10, indirectly counteracting oxidative stress through the FSP1-CoQ10 system (75). Cells overexpressing GCH1 were found to possess two polyunsaturated fatty acyl chains, a structural feature that significantly protects against the depletion of PUFAs in ferroptosis, serving as a physical defense mechanism against ferroptosis (76).

NAD(P)H:quinone oxidoreductase 1 (NQO1), an NAD(P)H-dependent quinone reductase similar to FSP1, can synergistically promote the conversion of ubiquinone to ubiquinol, inhibiting oxidation (77, 78). However, NQO1 is substrate dependent, and depending on the substrate, it may either promote or reduce ROS generation, suggesting that the inhibitory effect of NQO1 on oxidation may be unstable (79).

Therefore, as mentioned above, the FSP1-CoQ10 system clearly plays a crucial role in the antioxidant mechanism of ferroptosis by collaborating with multiple antioxidant systems, and this system holds significant research prospects.

#### 2.4.3 Other antioxidant systems

In the crucial xCT-GPX4 antioxidant system, Cys not only participates in the classic xCT-GPX4 pathway of antioxidation but also promotes the synthesis of sulfane sulfur (S°), thereby enhancing the antioxidative mechanism (80). Cys serves as the primary source of intracellular elemental sulfur (S (0)) and contributes to the biosynthesis of hydrogen sulfide, hydrogen polysulfides, and polysulfides, among other S° species (81). Among these, hydrogen sulfide, in particular, is a potent ROS scavenger capable of reducing lipid ROS generation to inhibit ferroptosis (80).

Furthermore, some mechanisms counteract ferroptosis by inhibiting the synthesis of lipid ROS. A previous study revealed that the gene encoding the phospholipid transporter SLC47A1 can be activated by the transcription factor peroxisome proliferator-activated receptor α (PPARA). And the activated PPARA-SLC47A1 pathway inhibits the production of esterified PUFAs, namely, cholesterol esters (CEs), to prevent lipid peroxidation in ferroptosis (82). Additionally, LOXs, a critical driver of lipid ROS formation, can be targeted to prevent ferroptosis. For example, research has shown that inhibiting ALOX15 in cancer-associated fibroblasts (CAFs) can block lipid ROS production, thereby suppressing ferroptosis (83).

### 2.5 Mammalian target of rapamycin

The mTOR pathway is a central regulator of cell growth, autophagy, and metabolism, comprising two distinct complexes: mTOR complex 1 (mTORC1) and mTOR complex 2 (mTORC2) (84). While mTORC2 is not well understood, mTORC1 has become a central regulator of cell metabolism, proliferation, differentiation, autophagy, and immune responses (85). The compositional differences between mTORC1 and mTORC2 influence their sensitivity to rapamycin, rapamycin have shown therapeutic potential, though combination therapies may be required to overcome resistanc (86). The phosphoinositide 3-kinase (PI3K)/protein kinase B (PKB, also called Akt)/mTOR (PAM) signaling pathway is activated by immune stimulation and is tightly regulated at multiple levels to prevent uncontrolled cellular proliferation (87). phosphatase and tensin homolog (PTEN), as a lipid phosphatase, dephosphorylates phosphatidylinositol-3,4,5–trisphosphate (PIP3) back to phosphatidylinositol-4,5-bisphosphate (PIP2), negatively regulating AKT signaling (87). Additionally, the tuberous sclerosis complex (TSC) acts as a negative regulator of mTORC1 by inhibiting ras homolog enriched in the brain (Rheb), a GTPase that activates mTORC1 (88, 89). adenosine 5’-monophosphate-activated protein kinase (AMPK) is activated due to low ATP levels, it inhibits mTORC1 both directly through phosphorylation and indirectly by activating the TSC (90, 91). Feedback loops within the pathway also ensure that mTORC1 activation suppresses upstream signaling to prevent excessive cell growth and maintain metabolic balance (87).

In LN, hyperactivation of mTOR signaling has been linked to glomerular damage, mesangial proliferation, and immune cell dysregulation (92). Recent studies show that cordyceps proteins (CP) modulate the mTOR pathway in LN, significantly reducing interleukin-6 (IL-6) and interleukin-1β (IL-1β) levels (93). Pharmacological inhibition of mTOR, such as with rapamycin, mycophenolate, etc., has demonstrated renoprotective effects in LN, underscoring its therapeutic potential (92, 94, 95). Research has also found that magniferin (MG) and Astragali Radix downregulate the mTOR pathway, thereby restoring T cell imbalance in LN (67, 96). These compounds may offer therapeutic potential by modulating the immune response and reducing inflammation in LN through their effects on mTOR signaling.

In LN, the activated T-cell receptor (TCR) regulates multiple metabolic pathways through mTOR, enabling T cells to undergo metabolic reprogramming from fatty acid oxidation and pyruvate oxidation metabolic patterns to glycolysis and glutaminolysis (97–99). During this process, changes associated with ferroptosis metabolism, such as increased lipid synthesis and ROS production (100), accumulation of labile iron (101), and weakened glutathione metabolism (102), occur within T cells (103, 104). Therefore, targeting the mTOR pathway in LN may not only help restore T cell balance but also reduce ferroptosis in T cells, providing a potential therapeutic strategy for managing LN (102).

### 2.6 NAD(P)H

NAD(P)H can serve as a biomarker for determining sensitivity to ferroptosis (105). The pentose phosphate pathway is the primary source of NAD(P)H, NAD(P)H plays a crucial role in ferroptosis regulation by providing reducing equivalents for antioxidant defense mechanisms (106). For instance, NAD(P)H provides hydrogen ions to convert cystine into cysteine, potentially influencing GSH production and promoting the xCT-GPX4 antioxidant system, which inhibits ferroptosis (107, 108). NAD(P)H functions through the FSP1-CoQ10-NAD(P)H pathway, alongside GPX4 and glutathione, to prevent phospholipid peroxidation (72). The study reveals that the mechanism of NAD(P)H in ferroptosis involves the membrane-associated RING-CH-type finger 6 (MARCHF6) E3 ubiquitin ligase in the transmembrane endoplasmic reticulum interacts with NAD(P)H through its C-terminal region, enhancing its activity and reducing ferroptosis (109). However, NAD(P)H can also induce ferroptosis. Electrons from NAD(P)H are transferred to oxygen by oxidoreductases, generating hydrogen peroxide, which then reacts with iron in the Fenton reaction, promoting ferroptosis (110). Therefore, NAD(P)H, as a key double-edged regulator of ferroptosis, plays a crucial role by supporting antioxidant defense mechanisms to suppress ferroptosis while also contributing to its occurrence through the generation of reactive oxygen species.

NAD(P)H-mediated ROS contributes to the immune imbalance observed in LN. Superoxide production, driven by NOX, is elevated in LN, contributing to the occurrence of ferroptosis (111). However, ROS from NOX are also involved in efferocytosis, enhancing the removal of dead cells and decreasing antigen production by influencing pH levels and proteolysis in efferosomes (112). Additionally, recent studies have demonstrated that NOX plays a role in SLE immunomodulation through its activity in the myeloid compartment and its selective inhibition of TLR7 signaling in B cells (113).

## 3 The role of T-cell ferroptosis in immune regulation

### 3.1 T-cell ferroptosis and immune regulation in cancer

**Figure 2:**
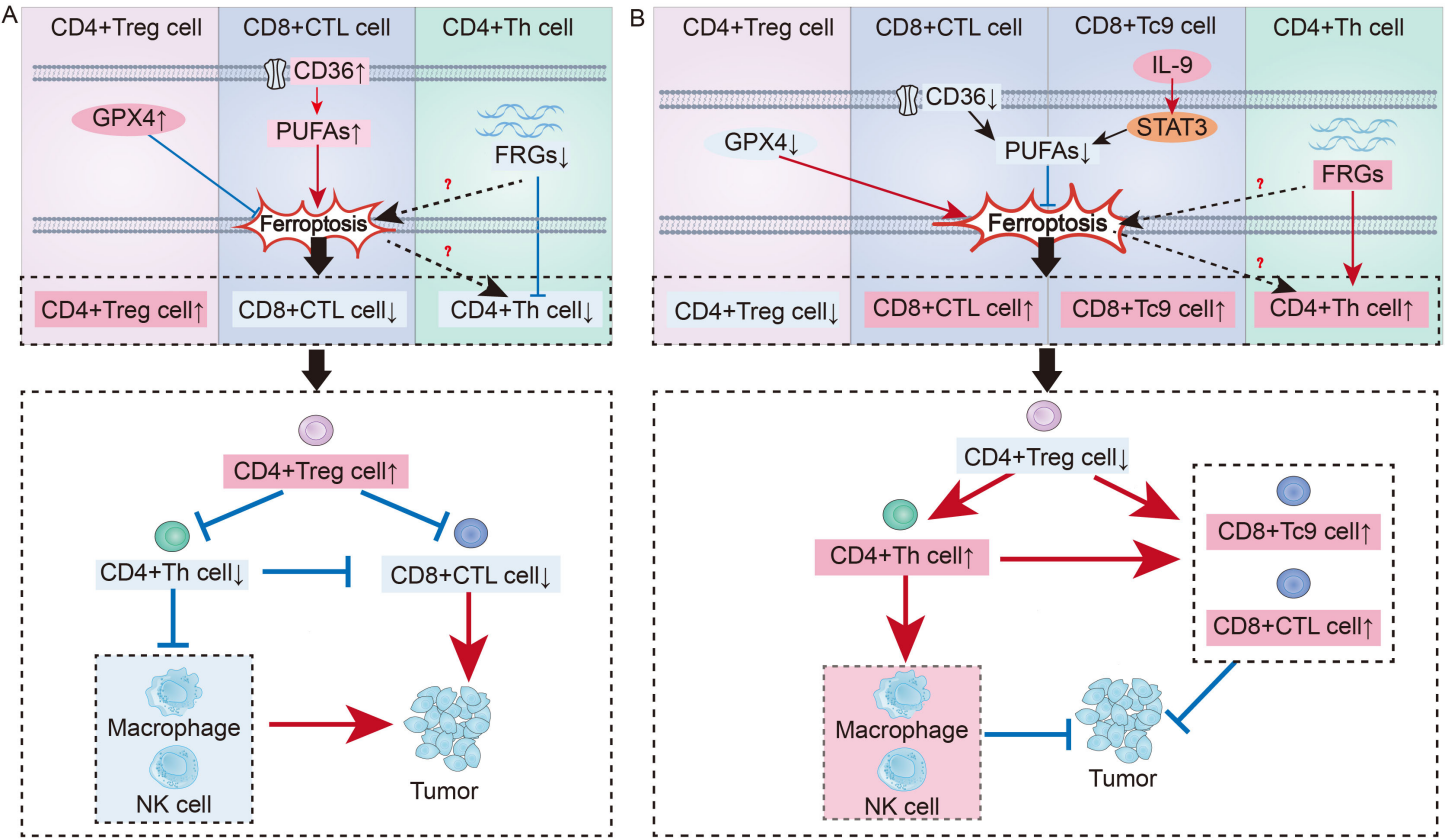
Immune regulation of T cell ferroptosis in cancer: **(A)** Mechanisms of T Cell Ferroptosis in Promoting Tumor Growth: In the tumor microenvironment, upregulation of Gpx4 in CD4+ Treg cells inhibits ferroptosis, leading to an increase in CD4+ Treg cells. Upregulation of the CD36 receptor on the surface of CD8+ CTL enhance intracellular PUFAs synthesis, promoting ferroptosis and resulting in a decrease in CD8+ CTL. Suppression of FRGs in CD4+ Th cells can promote ferroptosis, leading to a reduction in CD4+ Th cells. Thus, T cell immune imbalance regulated by ferroptosis contributes to tumor development. **(B)** Mechanisms by Which T Cell Ferroptosis Suppresses Tumor Growth: Downregulating Gpx4 in Treg cells can promote ferroptosis, increasing the number of CD4+ Treg cells. Blocking the CD36 receptor on CTL can inhibit ferroptosis, leading to an increase in CTL. Tc9 can suppress PUFAs synthesis through the IL-9-STAT3-fatty acid oxidation pathway, avoiding ferroptosis. After the suppression of FRGs in Th cells is lifted, it can promote an increase in Th cells. By reversing the T cell immune imbalance in the tumor environment, tumor development can be inhibited.

The immune regulatory mechanism associated with metabolic goals related to T-cell ferroptosis has attracted early interest in the area of tumor research (**Figure 2**). Inducing ferroptosis in CD8+ T cells is a vital tactic used by tumor cells to evade immune surveillance since it is the primary mechanism by which these cells are eliminated (114). CD8+ T cytotoxic (Tc) cells can be classified into many subtypes, such as Tc1, Tc2, Tc9, Tc17, and Tc22 cells. The Tc1 fraction is recognized as the conventional cytotoxic T lymphocyte (CTL) fraction and functions as the main effector subtype of CD8+ T cells (115). Furthermore, studies have shown that activated CD8+ T lymphocytes are highly susceptible to ferroptosis when they are present in the tumor microenvironment (TME) (116). Research has shown that upregulation of the fatty acid receptor CD36 on CTL results in increased production of PUFAs inside the cells. This, in turn, facilitates the ferroptosis of CTL (3, 117). By blocking CD36 or suppressing CD8+ T cell ferroptosis, it is possible to effectively restore their antitumor function.

Additionally, recent research has revealed the role of ferroptosis in other CD8+ T cell subsets in tumor immune regulation. Among the CD8+ effector T-cell subsets, Tc9 cells are characterized by IL-9 secretion (118). Although significantly fewer in number than CTL, they possess a tumor-killing capability per cell comparable to that of individual CTL (118). Notably, unlike CTL, Tc9 cells in the TME can upregulate fatty acid oxidation through the IL-9-STAT3-fatty acid oxidation pathway, reducing the accumulation of PUFAs and preventing ferroptosis (119). Given that Tc9 cells are less prone to ferroptosis in the TME than are CTL and have a higher survival rate, adoptive replenishment of Tc9 cells is expected to be a new therapeutic target for treating tumors.

In addition to enhancing the cytotoxic impact of CD8+ effector T cells, CD4+ Th cells also stimulate B cells and other effector cells to exhibit antitumor effects (120–122). Research has shown that in the gastric cancer TME, inhibiting the expression of ferroptosis-related genes (FRGs) in CD4+ Th cells weaken their activation, which is associated with poor tumor prognosis (123). Conversely, relieving the suppression of FRGs enhances the activation of CD4+ T cells in GC patients, improving the outcomes of immunotherapy. Therefore, ferroptosis in CD4+ Th cells is closely linked to immune imbalance, but the specific regulatory mechanisms require further investigation for clarification.

Furthermore, the high infiltration of Treg cells in cancer patients often indicates a low survival rate, as Treg cells play a key role in tumor immune evasion by helping tumor cells withstand antitumor immune responses (124). Several studies have shown that Treg cells in tumors exhibit increased GPX4, which is positively correlated with Treg cell survival (2, 125). Moreover, Treg cells lacking Gpx4 can not only induce ferroptosis in Tregs but also enhance the antitumor effect of Th17 cells through increased IL-1β, both of which are mechanisms that inhibit tumor progression (2). However, if lipid peroxides are further neutralized or iron chelators are used, Treg cells can regain their protumor survival state of high infiltration (2). Therefore, targeting Treg cell GPX4 or promoting ferroptosis in Treg cells could be key to improving tumor immunotolerance (**Figure 2**).

### 3.2 T-cell ferroptosis and immune regulation in other diseases

In recent years, the immune regulation of T-cell ferroptosis has also garnered attention in other diseases. Studies have shown that in autoimmune encephalomyelitis, inhibiting GPX4 promotes an excess of pathogenic T cells (126). Therefore, enhancing the xCT‒GPX4 axis or inhibiting other metabolic pathways that cause lipid peroxidation in pathogenic T cells could be a new therapeutic direction to improve the prognosis of patients with autoimmune diseases. Research has shown that during external pathogen infection, the xCT‒GPX4 antioxidant system helps to suppress the occurrence of ferroptosis in effector CD8+ and CD4+ T cells, promoting their expansion and enabling them to mount an immune response against pathogen invasion (4). Studies have also shown that by inhibiting mitochondrial ROS accumulation and promoting GPX4 effects, ferroptosis in memory CD4+ T cells can be suppressed, thereby contributing to long-term viral immune protection (127). In summary, metabolism related to T-cell ferroptosis and its key enzymes are critical targets for regulating immune balance in diseases and warrant further study.

## 4 Ferroptosis and T-cell immune imbalance in LN

LN is characterized by immune imbalance as an autoimmune disease. In LN, there is often an immune imbalance characterized by the upregulation of TFH cells, Teff cells and CD8+ effector T cells, along with the downregulation of Treg cells (128, 129). Inducing ferroptosis in B cells has been shown to reduce plasma cell formation and antibody production, greatly improving the prognosis of SLE (130). The cell debris produced by neutrophil ferroptosis is a stable source of autoantigens in SLE, and inhibiting neutrophil ferroptosis helps to alleviate SLE (70). However, recent studies provide a direct challenge to the concept that NETs promote autoimmunity and target organ injury in SLE (131).

**Figure 3:**
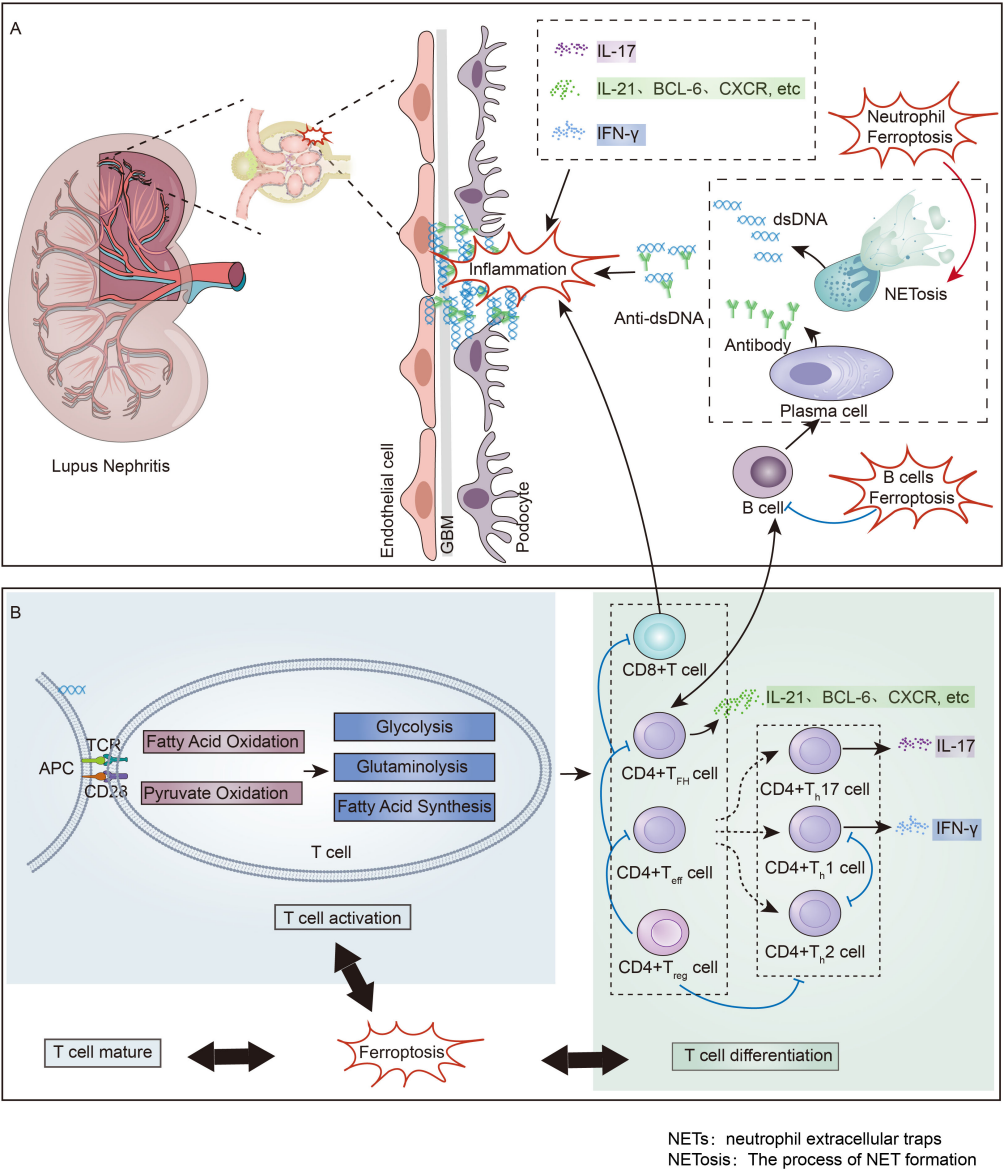
Immune Imbalance and Ferroptosis in Lupus Nephritis: **(A)** Non-T cell Immune Imbalance and Ferroptosis in Lupus Nephritis. Neutrophil ferroptosis induces the formation of NETs, which promotes the production of a large number of pathogenic antigens. These antigens stimulate B cells to activate into plasma cells and secrete antibodies, and the deposition of antibody-antigen complexes in the kidney leads to the onset of lupus nephritis. B cell ferroptosis has been found to suppress B cells, thereby inhibiting the occurrence of lupus nephritis. **(B)** T cell Immune Imbalance and Ferroptosis in Lupus Nephritis. Following the deposition of antibody-antigen complexes in the kidney, T cells in the kidney are promoted to become activated and differentiated, ultimately secreting various inflammatory factors that advance renal inflammation. Ferroptosis regulates the state of T cell immune imbalance by participating in the maturation, activation, and differentiation processes of T cells. NETs, neutrophil extracellular traps; NETosis, The process of NET formation.

T-cell ferroptosis also plays an immunoregulatory role has garnered significant attention. Research in LN patients has shown that iron accumulation within T cells promotes gene transcription by driving DNA hydroxymethylation and demethylation, thereby facilitating CD4+ T cell activation, which exacerbates lupus manifestations (132). Targeting excessive iron uptake in T cells could improve outcomes in SLE patients (133). Moreover, an *in vitro* study revealed that the maturation of peripheral T cells is closely related to T-cell ferroptosis (134). Research has shown that T-cell GPX4 deficiency can induce ferroptosis and inhibit T-cell proliferation (4). In the LN, to fulfill their corresponding effector functions, various differentiated T-cell subsets need to utilize all reprogrammed metabolic pathways to varying degrees (11, 135, 136). And the distinct metabolic pathway preferences of different T-cell subsets lead to changes in ferroptosis-related metabolism within their respective cells (137). Therefore, ferroptosis-related metabolism regulate abnormalities in the activation, development, maturation and differentiation of T cells in the LN (**Figure 3**).

Below, we discuss the connection between immune imbalance and ferroptosis-related metabolism in different T-cell subsets in the LN, including TFH, Treg, CD8+ T and Teff cells, and summarize the potential immunoregulatory mechanisms of T-cell ferroptosis in the LN.

### 4.1 TFH cell

Upon activation, naïve T cells differentiate into Teff in the LN, which are promptly dispatched to sites of inflammation to mount an immune response, whereas differentiated TFH cells remain within the lymph nodes or lymphoid follicles, specifically the germinal centers (GCs) (138). The characteristic phenotype of TFH cells includes the expression of C-X-C chemokine receptor type 5 (CXCR5), induced T cell costimulator (ICOS), programmed cell death protein 1 (PD-1), B-cell lymphoma 6 (Bcl-6), and interleukin-21 (IL-21), which are integral to regulating the differentiation of TFH cells and the formation of GCs (139). The interaction between CXCR5 and its ligand C-X-C chemokine ligand 13 (CXCL13) facilitates the migration of TFH cells to GCs and promotes sustained activation of B cells within GCs (140). BCL-6 is the master transcription factor for TFH cells, leading to the differentiation of naïve T cells into TFH cells. Moreover, BCL-6 plays a crucial role in activating and differentiating B cells and forming GCs by regulating various target genes involved in antigen-triggered calcium signaling within TFH cells (141). Upon binding to its receptor on TFH cells, the cytokine IL-21 upregulates the expression of BCL-6, CXCR5, and ICOS through the janus kinase (JAK)- signal transducer and activator of transcription (STAT) axis, indirectly promoting the migration of TFH cells and the production of pathogenic antibodies (139). Studies have shown that targeting the autocrine cytokine IL-21 in the TFH cells of mouse models of SLE can help suppress the proliferation and development of TFH and Th17 cells, providing a treatment for SLE (142). Therefore, the upregulation of TFH cells in the LN, through high expression of the aforementioned phenotypes, can promote the migration of TFH cells to GCs and foster sustained reciprocal stimulation between TFH cells and B cells within GCs (142–144). And the upregulation of TFH cells is a significant factor in the production of pathogenic antibodies in LN and accelerates the progression to end-stage renal disease in patients with LN (145).

Iron is an important element in promoting the normal differentiation of T cells and maintaining normal metabolism (146). Recent studies have shown that miR-21 overexpression in CD4+T cells promote iron accumulation by inhibiting 3-hydroxybutyrate dehydrogenase 2 (BDH2) in lupus-susceptible mice. Cellular iron accumulation can promote BCL6 gene hydroxymethylation by enhancing Fe2+-dependent TET enzyme activity in TFH cells, thereby promoting TFH cell differentiation (147). Given that the accumulation of intracellular iron not only is crucial for TFH cell differentiation but can also induce ferroptosis, whether the balance between these two factors can be modulated to improve TFH cell immune imbalance has attracted increasing attention (148).

**Figure 4:**
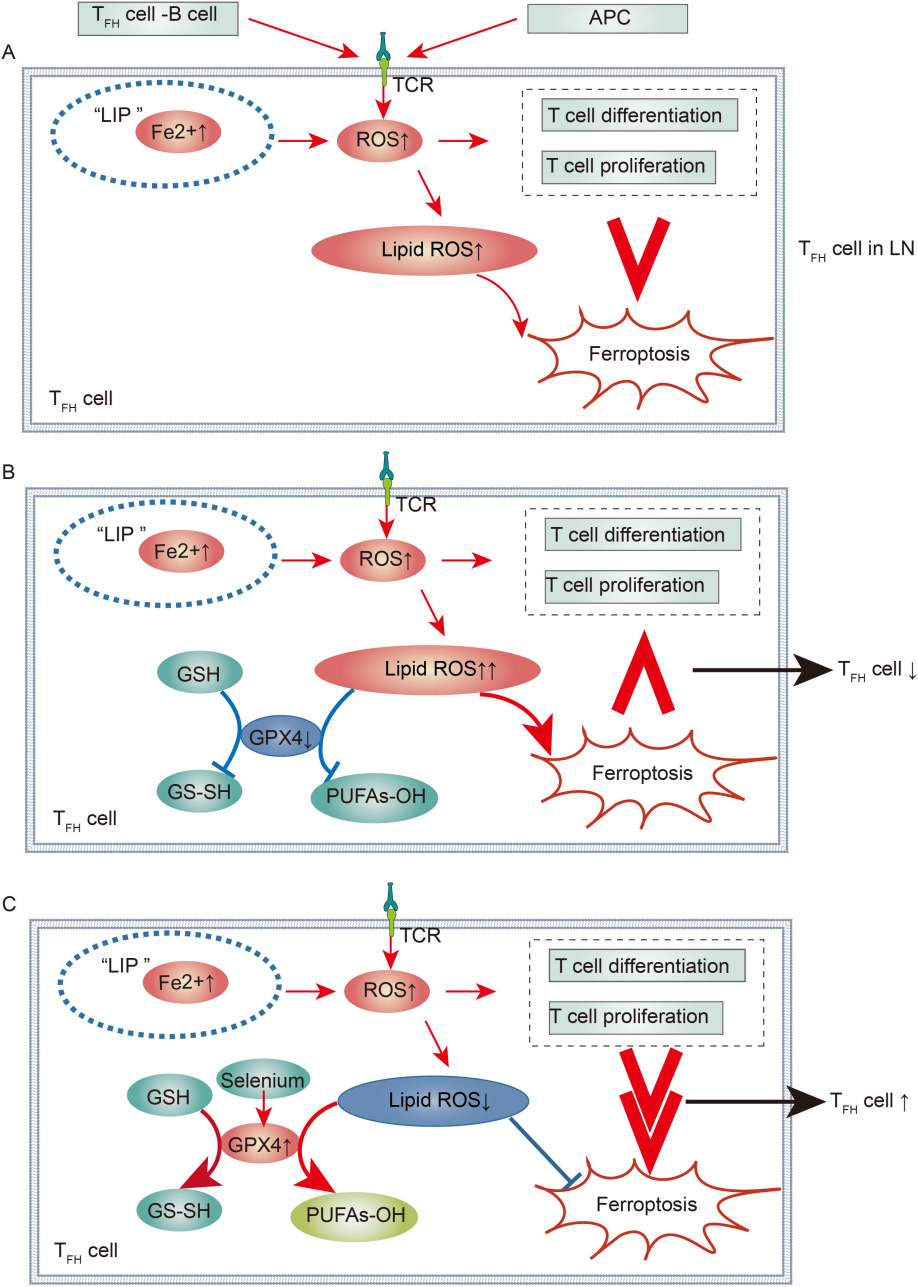
Immunological Regulation of Ferroptotic TFH Cells in Lupus Nephritis: **(A)** TFH Cells in Lupus Nephritis. In the context of lupus nephritis, the interaction between TFH and B cells, along with TCR stimulation activation, leads to a significant increase in ROS within TFH cells. Concurrently, there is an increase in Fe uptake within TFH cells. This not only promotes TFH cell differentiation and proliferation but also ferroptosis. However, the overall effect of ferroptosis is less than that of promoting differentiation and proliferation, resulting in a large number of TFH cells contributing to the progression of lupus nephritis. **(B, C)** Regulation of TFH Cell Immune Imbalance in Lupus Nephritis via the Selenium-GPX4-Ferroptosis Axis. By downregulating GPX4, ferroptosis can be promoted, making its effect greater than that of promoting differentiation and proliferation, leading to a reduction in TFH cells. Conversely, by increasing selenium to upregulate GPX4 activity, ferroptosis can be inhibited, thus diminishing its effect compared to differentiation and proliferation, resulting in an increase in TFH cells.

Upon TCR activation, there is a marked increase in intracellular ROS within T cells, which is primarily mediated by the mTOR pathway, which represents the predominant source of ROS (149). Studies have revealed that activated TFH cells contribute to elevated ROS levels not only through intrinsic TCR signaling but also through sustained interaction with B cells in GCs, thereby increasing ROS production (99, 150). Furthermore, these activated TFH cells accumulate lipid peroxides, and mitochondrial alterations are consistent with a ferroptosis phenotype (150). Advanced investigations have shown that specific deletion of GPX4 in mouse T cells accelerates the depletion of TFH cells and attenuates the TFH-B-cell interaction within GCs, leading to reduced production of pathogenic antibodies. Conversely, selenium supplementation has been found to reverse this trend (150). Thus, the selenium-GPX4-ferroptosis axis is a central regulator of TFH cell immune homeostasis. However, whether this axis can modulate immune imbalances caused by increased TFH cells in the LN remains to be confirmed by further research (**Figure 4**).

So far, clinical studies highlight the potential of targeting TFH cells for LN treatment. Recent clinical studies have shown that a CXCR5-directed antibody promotes the depletion of TFH cells in SLE, demonstrating its clinical potential for treating autoimmune diseases (151). Furthermore, follicular regulatory T (TFR) cells localize to the GC where TFH cells reside by expressing CXCR5 and FoxP3 and regulate their function (152). A clinical study found that PD-1 on TFR cells promotes hypermethylation in the CNS2 region of the FoxP3 gene, leading to reduced FoxP3 expression and impaired suppressive function (153). However, IL-2 supplementation therapy can restore this lost regulatory function *in vitro* (153). Notably, recent clinical studies have demonstrated the potential of low-dose IL-2 therapy in restoring TFR-TFH regulation *in vivo (* 154).

### 4.2 Treg cell

The primary function of Treg cells is to inhibit hyperactive pathogenic immune responses to maintain immune homeostasis. They exert their immunosuppressive effects by directly or indirectly targeting T cells and B cells, with the expression of Forkhead Box Protein 3 (FOXP3) being essential for sustaining immune tolerance (136). Studies in LN have identified deficiencies in both the number and function of Treg cells (155, 156). Treg cells highly express CD25, and IL-2 can bind to the IL-2 receptor (IL-2R) subunit CD25, which is also known as IL-2Rα (157). IL-2 stimulation is crucial for the maintenance of Treg cells and for the differentiation of CD4+ T cells into specific effector T cell subsets following antigen-mediated activation (158).

The metabolic environment in LN following Naïve T cell activation has been found to be detrimental to Treg cell survival, resulting in the downregulation of Treg cells (156). These changes are closely associated with ferroptosis-related pathways, suggesting that modulating ferroptosis could potentially enhance Treg cell survival and help restore immune balance (128). For instance, the survival and differentiation of Treg cells rely more on fatty acid oxidation and oxidative phosphorylation for energy production, necessitating a metabolic environment in Tregs that generates less lipid ROS (159). Furthermore, research has found that the high expression of thioredoxin-1 in human Treg cells counteracts the oxidizing effects of ROS, resulting in a low ROS environment that favors Treg cell survival (160). Therefore, inhibiting ferroptosis in Tregs appears to be conducive to the metabolic environment required for their proliferation and differentiation. Recent studies in SLE have shown that blocking the cell surface protein CD71 to reduce intracellular iron accumulation and inhibit ferroptosis promotes Treg proliferation (133). In cancer-related research, upregulation of GPX4 has been identified as a key mechanism to inhibit ferroptosis in Treg cells and promote their survival (2, 161). Hence, in LN, enhancing GPX4 activity or suppressing the metabolic processes that lead to lipid ROS production in Treg cells could likely be a critical mechanism to promote Treg proliferation and differentiation, representing an important target for improving LN prognosis.

A prospective study found that vitamin D treatment, currently used in LN therapy, is beneficial for increasing Treg cells in SLE patients (162). In SLE patients, Treg cell dysfunction has been found to result from a persistent decrease in Foxp3 expression in Tregs, mediated by the involvement of the OX40L/OX40 axis (163). In a recent Phase I trial, infusion of umbilical cord-derived mesenchymal stromal cells (MSCs) led to an increase in Treg cells in SLE patients, showing effectiveness in treating lupus (164, 165). Since CD25 is the high-affinity subunit of IL-2R, low-dose IL-2 preferentially expands Treg cells, while higher doses further stimulate effector T cells and NK cells (158). Phase II clinical trials have confirmed that low-dose IL-2 is beneficial for active SLE by upregulating Treg cells (166, 167). These researchs demonstrate the potential of Treg cell therapies for treating LN.

### 4.3 CD4+ Teff cell

In LN, an increased immune imbalance involving Teff cells, including Th17, Th1, and Th2 cells, is an important factor that promotes inflammatory immune responses (168). Among these factors, the significant increase in Th17 cells is a crucial pathogenic mechanism in the renal damage experienced by LN patients (169). The differentiation of Th17 cells in the LN requires the cytokines IL-6 and transforming growth factor β (TGFβ), which activate the lineage-defining transcription factor receptor–related orphan nuclear receptor γt (RORγt) through the JAK-STAT pathway (170–172). Furthermore, the cytokine interleukin-23 (IL-23) is vital for the expansion and survival of pathogenic Th17 cells in LN by activating the STAT-3 pathway (173). Inflammatory cytokines secreted by Th17 cells, such as interleukin-17A (IL-17A), interleukin-17F (IL-17F), and interleukin-22 (IL-22), are key factors that drive the progression of autoimmune kidney diseases (174). Several studies have shown that the Th17/IL-17 axis significantly contributes to structural and functional renal damage in lupus nephritis by fostering proinflammatory environments and activating profibrotic pathways through cytokine secretion (175, 176).

In healthy organisms, Th1 cells secrete interleukin-12 (IL-12) and interferon-gamma (IFN-γ) to exert cellular immunity to prevent bacterial and viral invasion (177). Th2 cells secrete interleukin-4 (IL-4), IL-6, and interleukin-4 (IL-10) to perform humoral immunity against helminths and other extracellular microbes (177). Research has shown that IFN-γ secreted by Th1 cells inhibits Th2-related functions, whereas IL-4 and IL-10 secreted by Th2 cells suppress Th1-related functions (178, 179). In LN, an increasing body of research has demonstrated that a Th1 differentiation advantage, which enhances cellular immune attack on healthy tissue cells, is involved in the progression of LN (180, 181). Moreover, inflammatory cytokines secreted by Th1 cells promote the formation of an inflammatory environment in the LN (182–184). Specifically, autocrine interleukin-12 (IL-12) from Th1 cells can promote self-differentiation of Th1 cells and, together with T-bet and the transcription factors STAT4 and STAT1, drive the production of the major inflammatory cytokine IFN-γ in lupus nephritis (185).

Research has revealed that in mammals, there are two types of mTOR protein complexes: mTORC1 and mTORC2 (186). mTORC1 promotes the differentiation of Th1 cells and Th17 cells, whereas mTORC2 mediates the differentiation of Th2 cells (187). In lupus patients, studies have shown that inhibiting the mTORC1 pathway contributes to disease outcome (92, 188). Therefore, the mTORC1 pathway is crucial for the pathogenesis of LN, and it promotes the formation of an immune imbalance characterized by the upregulation of Th17 cells and a Th1 cell differentiation advantage in LN. In LN, since the mTOR protein complex pathway is closely related to cellular metabolism, it affects various Teff cells, including the synthesis of PUFAs, ROS production, and other ferroptosis-related metabolic changes (189, 190). Thus, whether ferroptosis can regulate the Teff immune imbalance through ferroptosis metabolic targets is worth investigating.

In LN, the hyperactivated PI3K/Akt/mTOR pathway mediates high glycolytic metabolism and an elevated glutaminolysis pattern, which promotes the hyperactivity of synthetic metabolic processes such as protein, lipid, and carbohydrate metabolism within cells, fostering the differentiation and proliferation of Teff cells (128, 133). Studies have shown that the glucose transporter 1 (Glut1) is expressed on Teff cells, which can further increase the glycolytic metabolism of Teff cells (191). Further research revealed that calcium/calmodulin-dependent protein kinase IV (CaMK4) promotes the glycolytic process in Th17 cells by stimulating the Akt/mTORC1 pathway and upregulating Glut1 (192). Additionally, studies have shown that the mTOR pathway promotes the expression of hypoxia-inducible Factor 1α (HIF1α) in Th17 cells, enhancing glycolysis (193). Compared with other Teff cells, Th17 cells present high glycolytic metabolism, which significantly promotes iron death-related metabolic changes, such as increased ROS production and increased synthesis of PUFAs, which are positively correlated with the differentiation trends of each Teff cell type.

An *in vitro* study revealed that glutaminolysis is crucial for Th17 inflammatory diseases such as LN. Owing to the different dependencies of Teff cells on the glutaminolysis pathway, glutaminolysis promotes the differentiation of Th17 cells but constrains the differentiation of Th1 cells and CTLs (194). The glutaminolysis induced by mTOR leads to changes in the accumulation of ROS in Teff cells (191). Initially, the breakdown of glutamine in Teff cells yields glutamate. Glutamate can subsequently increase the synthesis of GSH to inhibit the production of ROS (195). Moreover, glutamate can be further metabolized to produce α-ketoglutarate (α-KG). α-KG can promote ROS generation through the tricarboxylic acid cycle to enhance the mTOR-mediated metabolic pathway, and it can also alter chromatin accessibility by affecting histone methylation, thereby promoting cell differentiation (196). Therefore, the differentiation of various Teff cells promoted by glutaminolysis is positively correlated with the accumulation of ROS within these cells.

In summary, the differentiation trends of Teff cells are positively correlated with the accumulation of ROS and PUFAs within these cells. The antioxidant gene nuclear factor erythroid 2-related factor 2 (NRF2) can significantly inhibit the differentiation of Th17 cells in LN by suppressing ROS, thereby improving prognosis, which also confirms this point (197). Notably, these metabolic processes not only promote the differentiation and proliferation of Teff cells but also facilitate the occurrence of ferroptosis. Moreover, recent studies have shown that the expression of the transferrin receptor CD71 on the surface of Teff cells in SLE is significantly increased, with noticeable accumulation of intracellular iron (133). Past research has indicated that different Teff cells have varying levels of labile iron stores, which may lead to inconsistent regulation of ferroptosis among these cells (198). Notably, recent studies have shown that activated CD4+ Teff cells do not exhibit the characteristic changes in lipid ROS deposition associated with ferroptosis as much as TFH cells do, which may be related to TFH cells having more sources of ROS (148, 150). Similarly, since Th17 cells accumulate more ROS and PUFAs than other Teff cells do, promoting an increase in lipid ROS or inhibiting antioxidant mechanisms seems to be more conducive to the occurrence of ferroptosis in Th17 cells. Promoting Th17 cell ferroptosis is a promising therapeutic direction for LN and warrants further investigation.

Studies have found that in SLE patients, CaMK4 inhibits the transcription of IL-17A and IL-17F through dual mechanisms, by suppressing the activation of CREMα and the AKT/mTOR pathway, thereby indirectly inhibiting Th17 differentiation (199). After 6 months of vitamin D supplementation in SLE patients, it not only has favorable clinical effects on SLE, but also produces beneficial immunological effects by promoting a decrease in Th1 and Th17 cells in these patients (162, 200). Ustekinumab is a monoclonal antibody targeting the shared p40 subunit of IL-12 and IL-23 (201). IL-12 promotes the differentiation of Th1 cells and the secretion of IFN-γ, while the IL-23/Th17 axis plays a key role in the development of lupus (202). After ustekinumab treatment in SLE patients, a significant reduction in the IFN-γ response was observed, but no modulation of Th17-related genes was detected (202). Currently, the potential of Teff cell-driven treatment strategies for LN is significant, but many aspects remain unexplored and require further investigation.

### 4.4 CD8+ T cell

In LN, an increase in CD8+ T cells suggest a poor prognosis (203, 204). Studies have shown that in patients with juvenile-onset SLE, there is a significant increase in total CD8+ T cells and naïve CD8+ T cells, whereas effector memory CD8+ T cells are decreased (205). A clinical study indicated that the expansion of CD8+ memory T cells was associated with a poor prognosis for patients with LN (206). In LN, classic CD8+ effector T cells, namely, CTL, have been found to have defects in their cytotoxic function. This not only promotes autoimmune hyperactivity but also facilitates the invasion of pathogens (207). Moreover, the potential effects of other nonclassical CD8+ effector T cell subpopulations in the LN are beginning to receive attention (208–210). For example, subpopulations with effects similar to those of Treg cells have been identified, and increasing their numbers may become a new treatment method for LN (208, 211). Research has also revealed that effector CD8+ T cells characterized by high expression of granzyme K (GzmK) and low expression of granzyme B (GzmB) and perforin have relatively weak cytotoxic effects, driving the development of LN inflammation through the secretion of cytokines (207, 212, 213). Therefore, molecules that regulate the immune balance of CD8+ T cell subpopulations and related signaling pathways are potential therapeutic targets for LN.

The mTORC1 pathway affects the response of CD8+ effector T cells, whereas mTORC2 activity regulates memory CD8+ T cells (214). Therefore, metabolic processes within CD8+ T cells in the LN promote ferroptosis caused by the accumulation of lipid ROS and enhance cell differentiation. Studies have shown that Gpx4 is a major factor for the survival of peripheral CD8+ T cells in the TME and that ferroptosis induced by GPX4 deficiency can limit the expansion of CD8+ T cells (4). Research has also demonstrated different sensitivities to ferroptosis among CD8+ T cell subpopulations (119). Thus, metabolic targets of ferroptosis, such as GPX4, are likely key in regulating the proportions of different CD8+ T cell subpopulations and suppressing the expansion of pathogenic CD8+ T cells in the LN. However, the regulatory mechanisms of ferroptosis in various subpopulations of CD8+ T cells in the LN are still under exploration and hold great promise.

In clinical studies of SLE patients, IFN-γ produced by CD8+ T cells is a key factor in enhancing indoleamine 2,3-dioxygenase (IDO) activity, which promotes the therapeutic effect of allogeneic MSCs in lupus (215). CD8+ T cells are the primary producers of IFN-γ in LN (216). Notably, the monoclonal antibody AMG 811, which targets IFN-γ, has demonstrated limited and transient effects in LN patients (217). Currently, the effects of mTOR inhibitors on CD8+ T cells have been identified in other diseases. For instance, Everolimus, an mTOR inhibitor, has been shown to significantly reduce the abundance and proliferation of CD8+ CD28- effector memory T (TEM) cell in post-kidney transplant patients, thereby decreasing the progression of inflammation (218, 219). Given the importance of mTOR in CD8+ T cells, clinical research on its role in CD8+ T cells remain lacking.

## 5 Targeted ferroptosis in the treatment of LN

### 5.1 Feasibility of targeting ferroptosis in LN patients

Targeting ferroptosis in LN could offer a novel and more precise approach compared to traditional therapies. One potential strategy involves the use of ferroptosis inhibitors, such as ferrostatin-1 and liproxstatin-1, which are known to prevent lipid peroxidation by inhibiting the enzyme system responsible for ferroptosis (220, 221). These inhibitors have been shown to attenuate kidney damage and experimental models of SLE, suggesting their potential for clinical application (70).

Another promising approach is the modulation of iron metabolism. Iron chelators, such as deferasirox, have been used in various diseases to reduce iron overload and prevent ferroptosis (222, 223). In the context of LN, iron chelation may help decrease iron overload, thereby reducing ferroptosis-associated kidney injury (68, 224, 225). Additionally, agents such as Erastin, sulfasalazine (SSZ), and BSO inhibit the xCT-GPX4 system (226), while allosteric GPX4 activators promote the xCT-GPX4 system (227). Extensive research in diseases like cancer has demonstrated the effectiveness of these approaches (226). Regulating the GPX4 pathways could provide another avenue for therapeutic intervention LN (228, 229).

### 5.2 Potential side effects and safety considerations

While targeting ferroptosis offers exciting therapeutic potential, there are several important considerations regarding safety and potential side effects. Ferroptosis inhibitors and iron chelators, although effective in preclinical studies, may have off-target effects that need to be carefully evaluated in clinical trials (230). For instance, ferroptosis inhibitors may interfere with the normal functioning of oxidative stress pathways, which play a critical role in cellular defense against pathogens and cancer (231). Inhibition of ferroptosis may impair the ability of immune cells to respond to infections or tumorigenic cells, potentially increasing susceptibility to infections or promoting tumorigenesis (232).

In addition, iron chelation may lead to iron deficiency, which can impair cellular functions, particularly in rapidly dividing cells such as those involved in immune responses and erythropoiesis (233–235). Chronic iron depletion may also result in adverse effects on other organs, including the heart and liver, leading to organ dysfunction (236, 237).

Therefore, a balanced approach to targeting ferroptosis in LN is essential. Therapeutic strategies should aim to specifically modulate ferroptosis in the kidney and immune cells without affecting other critical physiological processes. Careful monitoring of iron levels, ROS generation, and immune function will be necessary to avoid unwanted side effects (238).

### 5.3 Current state of therapeutic development

Currently, the development of ferroptosis-targeted therapies for LN is in its early stages, with most studies being conducted in preclinical models. However, several promising strategies are being explored, and early-phase clinical trials are underway. For instance, liproxstatin-1 have shown efficacy in treating autoimmune diseases models, including LN (70). Iron chelators are being evaluated for their ability to reduce albuminuria in LN, but their effects and potential side effects related to ferroptosis and iron metabolism have not yet been fully explored (225).

Furthermore, understanding the immune regulatory role of ferroptosis in LN is critical for optimizing therapeutic strategies. Ferroptosis has been shown to influence the activation and differentiation of immune cells, such as T cells and B cell, which are central to the pathogenesis of LN (130, 147). By modulating ferroptosis in these immune cells, it may be possible to not only mitigate kidney injury but also restore immune tolerance and reduce autoimmunity (133).

## 6 Conclusions

Lupus nephritis often manifests as an immune imbalance characterized by the upregulation of Teff cells and CD8+ effector T cells, alongside the downregulation of Treg cells, which is related to the abnormal differentiation of various T cells. We further discovered that the cellular metabolism that induces T-cell differentiation in LNs also leads to the accumulation of lipid ROS within each T-cell subset. We found that Treg cell differentiation in the LN is restricted, whereas the intracellular accumulation of lipid ROS promotes ferroptosis. The differentiation of Teff cells in LN and the accumulation of intracellular lipid ROS both exhibit varying degrees of promotion, with a positive correlation observed between these enhancements. Among these, TFH cells have a greater source of ROS, leading to greater accumulation of lipid ROS in activated TFH cells, followed by Th17 cells, with other Teff cells accumulating even less.

Given the close relationship between T-cell ferroptosis metabolic targets and the generation of lipid ROS, we have summarized the potential mechanisms of immunoregulation by ferroptosis metabolic targets in LN. We found that reducing the accumulation of lipid ROS in Treg cells can promote Teff differentiation and inhibit Teff ferroptosis, whereas enhancing the accumulation of lipid ROS in Teff cells can significantly promote Teff ferroptosis, an effect greater than its ability to promote Teff differentiation. Notably, we emphasize the central regulatory role of the selenium-GPX4-ferroptosis axis in the immune dysregulation of TFH cells.

Ferroptosis also plays a role in other autoimmune diseases, including rheumatoid arthritis (RA) and multiple sclerosis (MS), though the mechanisms differ from those in LN. In RA, iron accumulation in synovial fibroblasts and macrophages contributes to the inflammatory environment within the joints (239). Excess iron promotes ROS production, leading to oxidative stress, which damages joint tissues and accelerates disease progression (240). However, RA primarily involves joint inflammation and does not feature the same degree of systemic immune cell dysfunction as in LN (241). In MS, oligodendrocytes is the cells responsible for the formation of myelin sheaths in the central nervous system (242). Iron accumulation in oligodendrocytes contributes to cell death through ferroptosis, impairing myelin regeneration and promoting neurodegeneration (243). While ferroptosis is implicated in the pathogenesis of MS, the disease is more focused on central nervous system damage rather than systemic immune dysfunction. In diseases such as autoimmune thyroiditis, inflammatory bowel disease (IBD), and myasthenia gravis (MG), ferroptosis-related metabolism influences immune cell activation and inflammation (244). Compared to LN, where ferroptosis directly contributes to immune dysfunction and kidney damage, the role of ferroptosis in these conditions is less directly reported.

Ferroptosis in LN has already garnered significant attention from researchers. On one hand, ferroptosis in renal cells during inflammation promotes tissue damage and triggers regional inflammatory responses (68, 245). On the other hand, immune cells, particularly T cells, contribute to persistent inflammation by continuously releasing inflammatory cytokines and phagocytosing healthy renal cells, underscoring their pivotal role in sustaining inflammatory processes in LN (246, 247). Ferroptosis in immune cells has been found to contribute throughout the process (248, 249). Neutrophils ferroptosis contributing to the generation of autoantigens (70). And inducing ferroptosis in B cells has been identified as an optimal strategy to reduce sustained antibody production (130). Although there is no direct evidence linking ferroptosis in T cells to LN, changes in ferroptosis-related metabolic pathways in T cells—including ROS, PUFAs, iron, GPX4, and GSH (100–102)—mediated through the mTOR pathway indirectly suggest a critical role for T-cell ferroptosis in regulating T-cell imbalance in LN (250).

Currently, various novel ferroptosis modulators, such as mitochondrial-targeted nanodrug systems (251), have been used to induce or block ferroptosis. However, the treatment of ferroptosis in LN is still mainly at the animal experiment or limited case study stage. More clinical trials are needed in the future to verify their safety and efficacy. Based on the current understanding of ferroptosis-related metabolic changes in T cells, we propose that ferroptosis-targeted drugs, by modulating ROS, PUFAs, iron, GPX4, GSH, etc., could be more beneficial for targeting T cells. Additionally, since ferroptosis manifests differently in each T cell type, determining the appropriate dose and adjusting drug combinations for inhibiting or promoting ferroptosis in appropriate T cell subset will be a key focus for future research. Currently, there is still much to explore regarding T cell ferroptosis treatment in LN, which warrants further attention.

In summary, research on the role of T-cell ferroptosis in immune regulation within the LN is still in its early stages. A better understanding of this potential immune regulatory mechanism from the perspective of ferroptosis metabolism will undoubtedly lead to novel therapeutic concepts. Not only could this involve mitigating LN-induced tissue damage by inhibiting the differentiation and survival of Teff cells, but it could also enhance immune tolerance by increasing the number of Treg cells, allowing for a more targeted approach to treating LN. Targeting additional metabolic aspects of T-cell ferroptosis may represent a promising future direction for the treatment of immune imbalance in LN.
